# The impact of social media use on appearance anxiety among college students —the chain mediating effect of upward social comparison and envy

**DOI:** 10.3389/fpsyt.2026.1962984

**Published:** 2026-10-15

**Authors:** Yongjian Lin, Zhihao Li, Luling Li, Zhen Lv, Tianxiang Song, Xiujie Zhou, Jianchao Ni

**Affiliations:** 1 School of Arts, Ningbo City College of Vocational Technology, Ningbo, Zhejiang, China; 2 School of Aerospace Engineering, Xiamen University, Xiamen, Fujian, China; 3 School of Marxism, Xiamen University, Xiamen, Fujian, China; 4 Institute of Education, Xiamen University, Xiamen, Fujian, China; 5 International School, Ningbo City College of Vocational Technology, Ningbo, Zhejiang, China; 6 School of Film Television and Communication, Xiamen University of Technology, Xiamen, Fujian, China

**Keywords:** appearance anxiety, chain mediation effect, envy, social media use, upward social comparison

## Abstract

**Background:**

The widespread adoption of digital media has reshaped young people’s aesthetic experiences. The abundance of idealized and digitally enhanced appearance-related content on social media has made appearance anxiety increasingly prevalent among university students. However, the psychological pathways linking social media use to appearance anxiety require systematic examination. This study examined the relationship between social media use and appearance anxiety among university students and tested the chain mediating roles of upward social comparison and envy.

**Methods:**

Using convenience sampling, 1,640 university students in China completed the Social Media Use Intensity Scale, Appearance Anxiety Scale, Upward Social Comparison Scale, and Envy Scale. Confirmatory factor analysis, correlation analysis, and PROCESS Model 6 analysis with 5,000 percentile bootstrap samples were used to examine the relationship between social media use and appearance anxiety and the chain mediating effects of upward social comparison and envy. All regression equations controlled for gender, educational level, and average daily social media use time.

**Results:**

(1) Social media use was significantly and positively associated with appearance anxiety among university students. (2) Upward social comparison mediated the relationship between social media use and appearance anxiety. (3) Envy mediated the relationship between social media use and appearance anxiety. (4) Upward social comparison and envy showed a significant chain mediating effect in the relationship between social media use and appearance anxiety.

**Conclusion:**

This study provided preliminary cross-sectional evidence for an appearance-anxiety-specific comparison–envy pattern linking social media use to appearance anxiety among university students. The findings provide theoretical and practical implications for aesthetic education and media literacy education in higher education within the digital media environment. They may also help guide university students toward more rational aesthetic perceptions, reduced appearance anxiety, and improved physical and mental well-being.

## 1 Introduction

Highly visual social media has become a central setting in which university students encounter, display, and evaluate appearance. Filters, beauty enhancement, and curated self-presentation can repeatedly expose users to selectively polished appearance ideals, while visible reactions from others, such as likes and comments, can make appearance a recurrent basis of social evaluation (1–4). Unlike traditional mass media, social media combines exposure to idealized visual content with interpersonal feedback and active self-presentation, creating repeated opportunities for comparison and evaluation (1, 3, 4). Appearance ideals are therefore encountered not only as distant cultural messages but also as standards enacted and endorsed within students’ own peer networks. Appearance anxiety refers to persistent concern that one’s appearance will be judged negatively and may involve heightened attention to perceived deficiencies, uneasiness, and avoidance (5, 6). Existing research links social media use with appearance preoccupation and appearance-related anxiety (2, 7). Recent reviews and meta-analyses further suggest that body-image risks are especially evident for highly visual and appearance-focused use and for online upward comparison, rather than being captured fully by overall social media exposure (3, 8, 9). The tripartite influence model provides the primary theoretical framework for this association. It proposes that media, peers, and family shape body-image outcomes through two central processes: the internalization of sociocultural appearance ideals and appearance-related comparison (10). Internalization refers to adopting external appearance standards as personal criteria, whereas appearance-related comparison involves evaluating the self against those standards (10). Social media is especially relevant because commercial imagery, influencer content, and curated peer posts are encountered within the same interactive environment, while visible engagement metrics can signal peer endorsement and make narrow ideals appear both prevalent and socially rewarded (1, 11, 12). This formulation is consistent with experimental evidence that viewing idealized influencer imagery can increase body dissatisfaction and negative mood, partly through state appearance comparison (13). The framework also distinguishes the overall sociocultural association from more specific psychological processes. Exposure to idealized appearance content can make discrepancies between external standards and the self more salient, but exposure alone does not specify how a particular user interprets those standards (14, 15). Upward social comparison represents one cognitive response through which an external ideal becomes a judgment about one’s relative standing. When the comparison target is attractive, socially relevant, and apparently successful, the perceived discrepancy may be experienced as personally consequential, and the resulting unfavorable self-evaluation may then be accompanied by envy, an affective response to a self-relevant unfavorable comparison (16). For appearance anxiety specifically, the perceived discrepancy may be accompanied by persistent attention to perceived appearance-related “flaws” and repeated concern about others’ evaluations (6, 17). Recent Chinese evidence provides context-specific support: qualitative findings show that social media can heighten appearance anxiety by shaping aesthetic standards and comparative environments, while survey evidence links upward social comparison on social media with greater appearance anxiety (17, 18). In the present study, social comparison theory is therefore used more narrowly to explain upward comparison, while cognitive appraisal theory supports envy as an emotional correlate of a self-relevant discrepancy. From the perspective of aesthetic education, digital media forms an important part of young people’s aesthetic experiences and standards. Clarifying the psychological chain linking social media use to appearance anxiety is therefore important not only for young people’s mental health but also for understanding how aesthetic education in higher education can guide them to perceive, appreciate, and pursue beauty appropriately.

Social comparison theory is used here only to specify how individuals evaluate themselves against superior appearance targets (19). Within the integrated account, rather than social comparison theory alone, the variables occupy the following roles: social media use constitutes the informational environment in which young people are situated; upward social comparison represents the cognitive appraisal process that occurs within this environment; envy is the emotional experience arising from an unfavorable comparison; and appearance anxiety is a negative psychological manifestation associated with the interaction between cognition and emotion. Social comparison theory is retained only to explain the comparative cognitive process, not the main association between social media use and appearance anxiety, while cognitive appraisal theory helps frame envy as an emotion associated with a self-relevant discrepancy (20). Evidence has identified a comparison–envy pathway for broad outcomes such as depression and subjective well-being (21, 22), but it remains unclear whether this pattern extends to appearance anxiety. Recent theoretical work on body image similarly argues that social-media variables should be positioned as distinct predictors, mediators, or moderators within established sociocultural frameworks rather than treated as a unitary exposure (12). Accordingly, the present study examines upward social comparison and envy as distinct correlates linked in a chain mediation model within the tripartite-model account of the main association. The domain-specific contribution is to examine whether this comparison–envy pattern is also observed for appearance anxiety.

### 1.1 The tripartite influence model and the association between social media use and appearance anxiety

Social media use refers to the extent to which social media is integrated into daily life (23), whereas appearance anxiety concerns worry that one’s appearance will be negatively evaluated by others (6). The tripartite influence model proposes that media, peers, and family contribute to body-image outcomes through the internalization of sociocultural appearance ideals and appearance-related comparison (10). In the present study, the model is used primarily to explain why greater involvement in a social-media environment may be associated with greater appearance anxiety. Social media is not merely a measure of time online; it is a hybrid sociocultural setting in which commercial media, influencers, and peers circulate overlapping appearance ideals (1, 11). Recent reviews likewise highlight visually oriented platform features, appearance-focused activities, and comparison processes as important considerations beyond time online alone (3, 12). Commercial images can provide culturally recognizable standards, influencers can present those standards as lifestyles that appear achievable, and peer photographs can make the standards locally and socially relevant (1, 11). Likes, comments, follower counts, and other visible indicators of approval may further communicate which appearances receive attention and recognition (3, 4). Consequently, the same narrow ideal may appear both descriptively common and socially expected. Internalization converts such an external standard into a personal criterion for self-evaluation (10). Once users judge their ordinary appearance against this criterion, normal variation can be interpreted as inadequacy. This process may be relevant to appearance anxiety because the outcome involves not only dissatisfaction with the self but also anticipation that others will notice, judge, or reject perceived shortcomings (6, 17). Frequent posting, editing, and monitoring of feedback may reinforce this evaluative orientation by encouraging users to view appearance as a public performance (2–4). Evidence linking appearance-focused social media activity with body-image concerns and appearance-rejection sensitivity is consistent with this account (1, 2, 4). More specifically, repeated exposure may normalize narrow appearance standards, while posting, editing, and visible feedback may heighten appearance focus and concern about others’ evaluations (1–4). Together, internalization, perceived social endorsement, and evaluative self-presentation explain the main association at a sociocultural level. This account differs from treating upward comparison as an all-purpose explanation: the tripartite influence model identifies the source and social authority of appearance pressure, whereas social comparison theory is reserved for the more specific cognitive process through which users position themselves relative to salient targets. The distinction also clarifies why the overall association may persist even after upward comparison and envy are included in the statistical model. Other processes implied by the tripartite framework, particularly internalization and perceived pressure, may remain relevant even though they were not directly measured. Accordingly, the following hypothesis was proposed:

H1: Social media use is significantly and positively associated with appearance anxiety among university students.

### 1.2 The mediating role of upward social comparison

Upward social comparison refers to evaluating oneself against someone perceived as superior on a personally relevant dimension (19). In this study, social comparison theory is used only to explain this cognitive pathway, not the main association between social media use and appearance anxiety. Social media makes superior targets unusually accessible: curated images of peers and influencers are frequent, visually salient, and often digitally enhanced. The relevance of these targets matters because users may know them personally, share a similar age or social position, or regard their lifestyles as attainable. These similarities can make the target a more diagnostic standard for self-evaluation than a distant celebrity. At the same time, selective presentation means that users often compare their ordinary, continuously experienced selves with other people’s most favorable moments. The available reference group can therefore become skewed toward unusually polished appearances, making exceptional appearances seem ordinary and intensifying perceived discrepancies between the self and an idealized target (14, 24). Repeated comparison may then narrow attention toward features on which the self appears deficient, while discounting the artificial production of the target image. Evidence that appearance comparisons with peers and influencers are associated with body dissatisfaction further supports the relevance of this pathway (11, 15). More recent evidence strengthens this account. A meta-analysis of 48 experiments found that exposure to upward comparison targets on social media produced a small but reliable negative effect on body image and related self-evaluations (9), while a 2025 meta-analysis of 83 studies found a moderate association between online social comparison and greater body-image concerns (8). Thus, greater social media use may be associated with more frequent and personally consequential upward comparison, which in turn may be associated with stronger negative evaluation of one’s own appearance and greater concern about how that appearance will be judged. Accordingly, the following hypothesis was proposed:

H2: Upward social comparison mediates the relationship between social media use and appearance anxiety among university students.

### 1.3 The mediating role of envy

Envy is a painful emotion that can arise when another person’s advantage is appraised as relevant to one’s own goals and self-worth (16, 20). This appraisal-based account is important because exposure to an attractive person does not automatically produce envy. The emotional response may depend on whether the target’s advantage is valued, whether it highlights a personally important shortfall, and whether the user interprets the discrepancy as attainable or difficult to control (16, 20). Contemporary envy research further emphasizes that envy is not motivationally uniform: benign envy is associated with upward motivation and self-improvement, whereas malicious envy is more closely associated with hostility toward the advantaged other (25). In appearance-focused social media settings, curated images can make attractive targets both salient and personally relevant. The discrepancy between a desired appearance and a seemingly superior target may therefore be experienced not only as a cognitive comparison but also as an emotionally charged indication that one lacks a valued attribute (16, 20). Although envy can motivate self-improvement when the advantage appears attainable, it still contains painful awareness of relative disadvantage (25–27). When the ideal is digitally enhanced, repeatedly encountered, or resistant to personal effort, that discomfort may be sustained rather than resolved. Because envy maintains attention on what the other person possesses and what the self appears to lack, it may be accompanied by tension, self-doubt, repeated mental review of the comparison, and heightened monitoring of one’s own appearance (26, 28). In the appearance domain, this affective focus may increase sensitivity to signs of social evaluation and concern that perceived inadequacies are visible to others. Accordingly, social media use may show an indirect association with appearance anxiety through envy, and the following hypothesis was proposed:

H3: Envy mediates the relationship between social media use and appearance anxiety among university students.

### 1.4 The chain mediating roles of upward social comparison and envy

Upward comparison can make the discrepancy between the self and a superior target both cognitively clear and emotionally salient. From a cognitive-appraisal perspective, envy becomes more likely when that discrepancy concerns personally important goals or self-worth (20). Appearance is especially self-relevant in visually oriented social media environments, where idealized images of peers, influencers, and celebrities are readily available and where users’ own images can also become objects of public evaluation. The proposed chain therefore involves a shift from sociocultural information to personal cognition and then to affective concern. Social media supplies repeated appearance standards; upward comparison translates those standards into an assessment of where the self stands; and envy gives the perceived disadvantage motivational and emotional significance. Envy may then sustain attention to the advantages possessed by the target and to attributes that individuals believe they lack. This continued focus can make perceived appearance deficiencies more salient and increase concern about how those deficiencies will be evaluated by others. The chain pathway is theoretically distinguishable from the two parallel indirect paths. An upward comparison may be associated with appearance anxiety without producing strong envy, and envy may also arise from social-media content without an explicitly recalled comparison episode. The ordered path tests the additional possibility that comparative cognition and emotional response operate in sequence. Previous research has reported an ordered association among upward comparison, envy, and broader outcomes such as depression (22), but whether the same cognitive–emotional pattern extends to appearance anxiety remains unclear. The present study therefore treats social media use as the sociocultural context, upward comparison as the comparative cognition, envy as the affective response, and appearance anxiety as the domain-specific social-evaluative outcome. Accordingly, the following hypothesis was proposed:

H4: Upward social comparison and envy form a chain mediation pathway in the relationship between social media use and appearance anxiety among university students.

In summary, the tripartite influence model provides the primary account of the main association by locating social media within a sociocultural system of media and peer appearance pressures. It explains why repeated participation in that environment may be associated with appearance anxiety through the normalization, endorsement, and internalization of appearance ideals. Social comparison theory and cognitive appraisal theory are used more narrowly to specify the comparative and affective correlates within that environment. Previous research has usually examined these links separately or focused on broader outcomes such as depression and general well-being. The present study therefore tests whether upward social comparison and envy show separate indirect associations and a chain mediation pattern within a model of appearance anxiety among university students (**Figure 1**). This organization preserves the tripartite influence model as the overarching framework while using the other theories to clarify particular parts of the proposed process.

**Figure 1:**
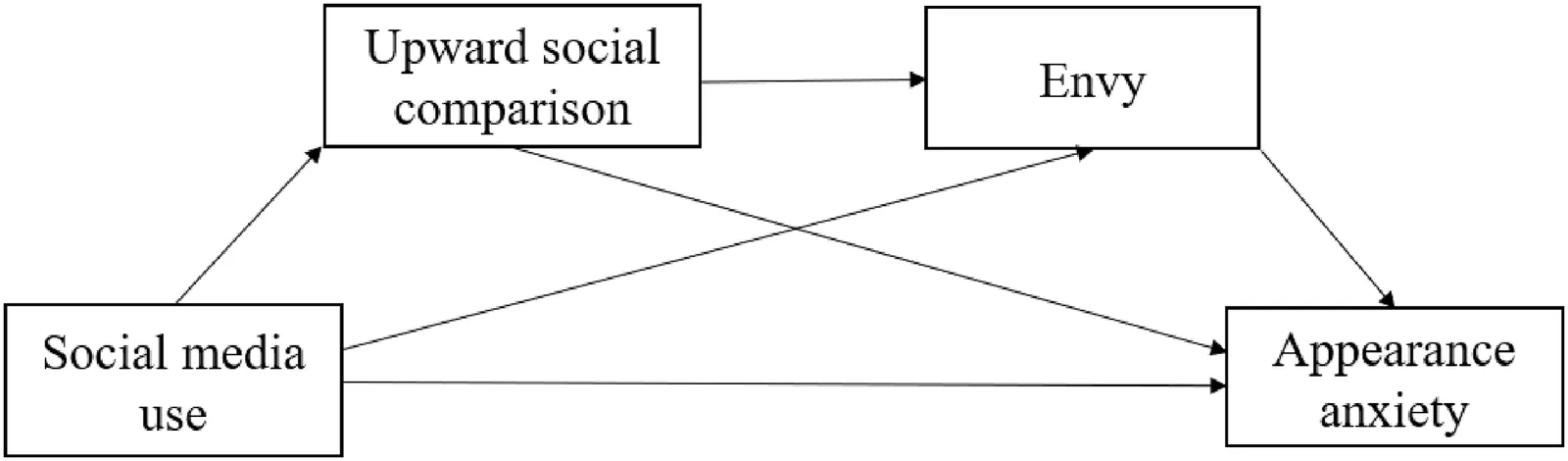
Chain mediating model.

## 2 Methods

### 2.1 Participants and procedure

This cross-sectional study used convenience sampling to administer a single-wave online questionnaire to university students from more than 20 institutions across eastern, central, and western China. The participating institutions represented different types and levels of higher education. These institutions served as the recruitment channels, through which the online questionnaire was circulated to eligible students. All measures were completed online at one time point. The study was approved by the Ethics Committee of Xiamen University (Approval No. XDYX202510K55). The first page of the online questionnaire presented the study purpose, voluntary nature of participation, anonymity protections, and the right to withdraw without adverse consequences. Students who provided informed consent proceeded to the questionnaire and completed the demographic and study measures in the same survey session. Submitted questionnaires were then screened using the quality criteria described below. A total of 2,000 questionnaires were collected. After 360 invalid questionnaires were excluded because of excessively short response times, logically inconsistent responses, or multiple missing responses, 1,640 valid questionnaires remained, yielding an effective response rate of 82%. Demographic information, including gender and educational level, was collected to describe the sample. Specifically, 936 participants were men (57.1%) and 704 were women (42.9%); 1,061 were undergraduate or associate-degree students (64.7%), 387 were master’s students (23.6%), and 192 were doctoral students (11.7%). The sample therefore included participants of different genders and educational levels.

### 2.2 Measures

All four measures used a 5-point Likert response format. Each construct score was calculated as the mean of its items, with higher scores indicating higher levels of the corresponding construct. The sources, numbers of items, and reliability and validity indices of the measures are described below. Complete items are provided in Appendix A.

#### 2.2.1 Social media use scale

The Facebook Intensity Scale developed by Ellison et al. (29) was adapted for the present study. The adapted scale contained 6 items. The KMO value was 0.839, and Cronbach’s α was 0.900. Confirmatory factor analysis yielded a composite reliability (CR) of 0.902 and an average variance extracted (AVE) of 0.607. Standardized factor loadings ranged from 0.662 to 0.892, with a mean of 0.775, indicating good internal consistency and convergent validity. In the adapted items, ‘Facebook’ was replaced by the social media app used most often, so the measure captured general engagement and integration into daily life.

#### 2.2.2 Appearance anxiety scale

The Social Appearance Anxiety Scale (SAAS), developed by Hart et al. (6), was used to assess anxiety arising from concern that one’s overall appearance would be negatively evaluated by others. The scale contained 10 items. The KMO value was 0.949, and Cronbach’s α was 0.951. Confirmatory factor analysis yielded a CR of 0.952 and an AVE of 0.664. Standardized factor loadings ranged from 0.750 to 0.901, with a mean of 0.813, indicating good internal consistency and convergent validity.

#### 2.2.3 Upward social comparison scale

A 6-item upward social comparison measure was developed by combining items from the online social comparison questionnaire developed by Steers et al. (30) and the social comparison orientation scale developed by Gibbons and Buunk (31). The KMO value was 0.908, and Cronbach’s α was 0.937. Confirmatory factor analysis yielded a CR of 0.938 and an AVE of 0.718. Standardized factor loadings ranged from 0.762 to 0.892, with a mean of 0.846, indicating good internal consistency and convergent validity.

#### 2.2.4 Envy scale

The envy measure used in this study was developed from the Dispositional Envy Scale by Lange and Crusius (26) and contained 5 items. The KMO value was 0.868, and Cronbach’s α was 0.909. Confirmatory factor analysis yielded a CR of 0.911 and an AVE of 0.672. Standardized factor loadings ranged from 0.717 to 0.878, with a mean of 0.818, indicating good internal consistency and convergent validity.

### 2.3 Data analysis

SPSS 26.0 and AMOS 26.0 were used for descriptive statistics, correlation analysis, and confirmatory factor analysis to evaluate scale reliability, discriminant validity, and correlations among the study variables. The PROCESS macro developed by Hayes (32) was used to test the chain mediating model, and percentile bootstrap confidence intervals were calculated for the indirect effects. The control variables were gender, educational level, and average daily social media use time, and they were entered in all regression equations. Gender and educational level were included to account for basic differences in sample composition, while daily use time was included to distinguish exposure duration from the focal measure of social media integration. These analyses were used to test the study hypotheses. Consistent with the cross-sectional design, the chain mediation estimates are interpreted as statistical indirect effects (33).

## 3 Results

### 3.1 Test of common method bias

Because data were collected using self-report measures, common method bias was possible. Procedural controls included emphasizing anonymity and confidentiality and including reverse-scored items. Confirmatory factor analysis was then used to examine common method bias. The one-factor model showed poor fit, χ² = 13,832.911, df = 324, p < 0.001, RMSEA = 0.159, TLI = 0.647, and CFI = 0.674, indicating that a single common factor did not adequately account for the covariance (34).

### 3.2 Confirmatory factor analysis and correlation analysis

Confirmatory factor analysis (CFA) was conducted in AMOS 26.0 to evaluate the measurement validity of the primary variables. As shown in **Table 1**, all standardized factor loadings met acceptable criteria, Cronbach’s α and composite reliability (CR) values exceeded 0.70, and average variance extracted (AVE) values exceeded 0.50, indicating good internal consistency and convergent validity. The square root of the AVE for each variable was also greater than its correlations with the other variables, indicating acceptable discriminant validity. A more stringent heterotrait-monotrait (HTMT) analysis was also conducted. As shown in **Table 2**, the HTMT ratios ranged from 0.678 to 0.749, and all were below the conservative 0.85 criterion, providing additional support for discriminant validity (35).

**Table 1:** Descriptive statistics, reliability, validity, and correlations among the study variables.

Variable	*M*	*SD*	1	2	3	4	CR	AVE	CFA loading range (mean)
1. Social media use	3.168	0.984	0.779	—	—	—	0.902	0.607	(0.662–0.892, 0.775)
2. Appearance anxiety	2.878	0.919	0.642***	0.815	—	—	0.952	0.664	(0.750–0.901, 0.813)
3. Upward social comparison	2.959	0.962	0.693***	0.736***	0.847	—	0.938	0.718	(0.762–0.892, 0.846)
4. Envy	3.089	0.933	0.680***	0.736***	0.727***	0.820	0.911	0.672	(0.717–0.878, 0.818)

* p < 0.05. ** p < 0.01. *** p < 0.001. Diagonal values are the square roots of the average variance extracted (AVE).

**Table 2:** Heterotrait-monotrait (HTMT) ratios among the study variables.

Variable	1	2	3	4
1. Social media use	—			
2. Appearance anxiety	0.682	—		
3. Upward social comparison	0.749	0.741	—	
4. Envy	0.678	0.746	0.736	—

All off-diagonal HTMT ratios were below the conservative 0.85 criterion (range = 0.678-0.749).

Correlation analysis showed significant positive correlations among social media use, appearance anxiety, upward social comparison, and envy. Specifically, social media use was positively correlated with envy (r = 0.680, p < 0.001), appearance anxiety was positively correlated with upward social comparison (r = 0.736, p < 0.001) and envy (r = 0.736, p < 0.001), and upward social comparison was positively correlated with envy (r = 0.727, p < 0.001). Social media use was also positively correlated with appearance anxiety (r = 0.642, p < 0.001) and upward social comparison (r = 0.693, p < 0.001). These correlations provided preliminary support for testing the mediating effects. Because several variables were highly correlated, collinearity diagnostics were conducted before the mediation analysis. Variance inflation factors for all predictors (2.037, 2.066, and 2.361) were below 5, indicating no serious multicollinearity (36).

### 3.3 Testing the hypothesized model

Chain mediation analysis examines indirect pathways through which an independent variable is statistically associated with a dependent variable via multiple ordered mediators. Path coefficients are estimated using multiple regression, and the significance of indirect effects is evaluated using their confidence intervals (37). In the present study, the PROCESS macro was used to test chain mediation involving upward social comparison and envy in the relationship between social media use and appearance anxiety. The 95% confidence intervals (CIs) for the indirect effects were estimated using 5,000 bootstrap samples. An indirect effect was considered significant when its 95% CI did not include zero (38). The results are presented in **Tables 3** and **4** and **Figure 2**. The control variables were gender, educational level, and average daily social media use time. In this cross-sectional design, the estimates quantify statistical indirect effects and do not establish temporal precedence or causal mediation (33).

**Table 3:** Regression equations for the chain mediating model.

Outcome variable	Predictor	*R* ^2^	*F*	B	*t*
Upward social comparison	Constant	0.4910	394.3079***	1.4266***	9.7835
	Social media use			0.6747***	35.4026
Envy	Constant	0.5281	365.7785***	1.5162***	10.8208
	Social media use			0.2982***	12.6041
	Upward social comparison			0.4313***	18.6728
Appearance anxiety	Constant	0.4093	283.2393***	1.6595***	11.0515
	Social media use			0.5700***	29.0446
Appearance anxiety	Constant	0.5990	406.4927***	0.4342**	3.2926
	Social media use			0.1406***	6.2423
	Upward social comparison			0.3235***	13.9856
	Envy			0.3584***	15.9352

B = unstandardized regression coefficient. * p < 0.05. ** p < 0.01. *** p < 0.001.

**Table 4:** Bootstrap estimates of indirect effects.

Indirect effect	Effect	BootSE	95% bootstrap CI
Total indirect effect	0.4294	0.0229	[0.3854, 0.4747]
Ind1: Social media use -> upward social comparison -> appearance anxiety	0.2182	0.0232	[0.1736, 0.2644]
Ind2: Social media use -> envy -> appearance anxiety	0.1069	0.0146	[0.0796, 0.1362]
Ind3: Social media use -> upward social comparison -> envy -> appearance anxiety	0.1043	0.0129	[0.0798, 0.1303]

Effects were estimated using 5,000 percentile bootstrap samples. The confidence intervals are interpreted as cross-sectional statistical indirect effects.

**Figure 2:**
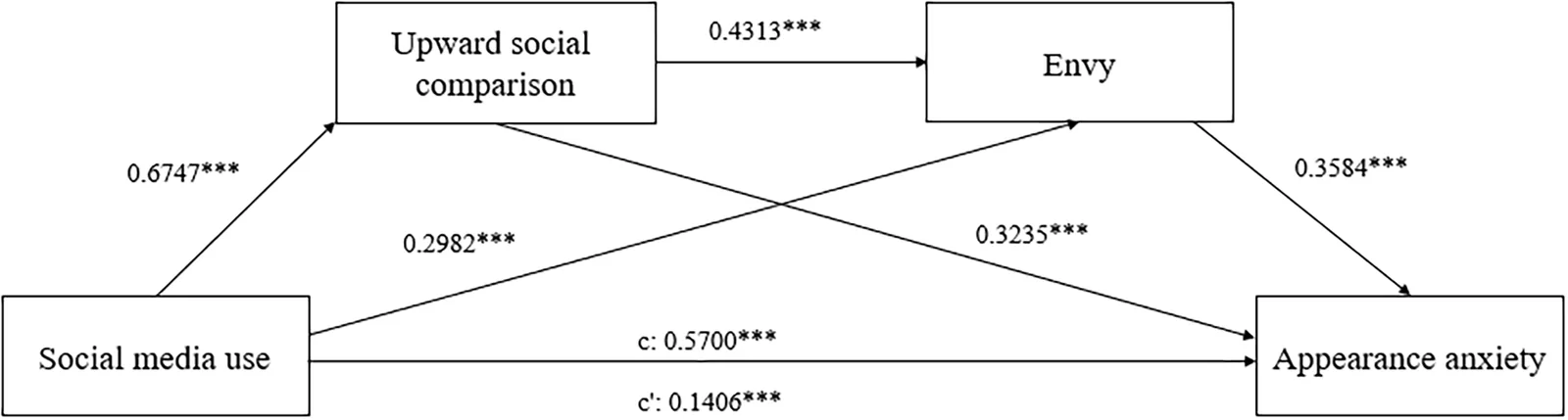
Path coefficients for the chain mediating model. *** p < 0.001.

Social media use was significantly and positively associated with appearance anxiety (B = 0.5700, SE = 0.0196, 95% CI [0.5315, 0.6085], p < 0.001), supporting H1. The total effect of social media use on appearance anxiety was significant. After upward social comparison and envy were included as mediators, the direct effect of social media use on appearance anxiety remained significant (B = 0.1406, SE = 0.0225, 95% CI [0.0964, 0.1848], p < 0.001), indicating partial mediation. The total indirect effect was 0.4294, accounting for 75.333% of the total effect. The total indirect effect comprised the following three pathways:

1. Ind1: Social media use → upward social comparison → appearance anxiety (BootSE = 0.0232, 95% bootstrap CI [0.1736, 0.2644]); because the interval did not include zero, the indirect effect was significant (effect = 0.2182), supporting H2.
2. Ind2: Social media use → envy → appearance anxiety (BootSE = 0.0146, 95% bootstrap CI [0.0796, 0.1362]); because the interval did not include zero, the indirect effect was significant (effect = 0.1069), supporting H3.
3. Ind3: Social media use → upward social comparison → envy → appearance anxiety (BootSE = 0.0129, 95% bootstrap CI [0.0798, 0.1303]); because the interval did not include zero, the indirect effect was significant (effect = 0.1043), supporting H4.

## 4 Discussion

Guided primarily by the tripartite influence model, this study examined the association between social media use and appearance anxiety and used social comparison theory and cognitive appraisal theory more narrowly to interpret the roles of upward comparison and envy. Social media use was positively associated with appearance anxiety, while upward social comparison and envy showed separate indirect associations and a chain mediation pattern. These patterns remained after adjustment for gender, educational level, and average daily social media use time. Because the data are cross-sectional, the discussion focuses on plausible sociocultural, cognitive, and affective explanations.

### 4.1 Social media use and appearance anxiety

The findings provided support for H1, indicating that social media use was positively associated with appearance anxiety. This interpretation is consistent with recent work highlighting visually oriented, appearance-focused engagement as particularly relevant to body-image concerns beyond generic measures of social-media use (3), and with qualitative findings among Chinese university students showing that social-media aesthetic standards and comparison climates can amplify appearance anxiety (18). The tripartite influence model provides the primary explanation for the main association. Social media combines media-generated, influencer-generated, and peer-generated appearance ideals within a shared sociocultural environment (10). This convergence matters because each source gives the ideal a different kind of authority: commercial media presents it as culturally desirable, influencers demonstrate and market practices for approximating it, and peers make it relevant to everyday social belonging. Repeated exposure can make a narrow appearance standard seem descriptively normal, while visible approval can make conformity appear socially valued. These signals may encourage users to internalize the ideal as a personal criterion even when they recognize that individual images are edited. When external standards are used to evaluate the self, ordinary discrepancies can become a basis for self-criticism and anticipated negative evaluation. The association with appearance anxiety is therefore understandable without assuming that every instance of social media use triggers an explicit comparison. The broader environment can shape what users believe a socially acceptable appearance should be, linking social media use with appearance anxiety. From a social-norm perspective, commercial images, influencer posts, and peer photographs appear side by side; their convergence may make the same ideal seem both culturally authoritative and socially typical. A second explanation concerns evaluative self-presentation. Social media users are simultaneously viewers and visible targets of evaluation; posting and editing images can turn appearance from a private attribute into a publicly displayed basis for feedback. The possibility of being viewed by peers may promote anticipatory monitoring before posting and repeated checking after posting. Quantified reactions can also make social approval appear measurable, encouraging users to interpret limited engagement or unfavorable comments as information about their appearance. Even when explicit negative feedback is absent, uncertainty about how an image will be received may maintain concern about external judgment. This platform-level account is distinct from the upward-comparison process discussed below. Accordingly, the association should be understood as sensitive to content and platform affordances, not as evidence that time online is inherently harmful. Empirical findings on appearance-focused use and appearance preoccupation are consistent with this interpretation (2, 4, 7).

### 4.2 The mediating effect of upward social comparison

The findings also supported H2, indicating a significant indirect association between social media use and appearance anxiety through upward social comparison. This indirect association represents a specific cognitive pathway rather than an explanation for the overall association between social media use and appearance anxiety. Beyond this theoretical description, an attention-selection and comparison-opportunity perspective helps explain the observed relationship. Social media lowers the practical cost of comparison: a simple swipe provides access to large numbers of selectively presented and digitally enhanced images. Repeated access can turn comparison from an occasional response into a habitual form of self-evaluation. Platform feeds also remove many contextual cues that would ordinarily qualify a comparison, such as the effort, selection, lighting, editing, or unsuccessful attempts behind an image. Users may therefore treat a highly managed presentation as direct evidence of another person’s ordinary appearance. The resulting reference group may therefore be systematically skewed toward unusually polished appearances, making an idealized standard seem ordinary rather than exceptional. Because this reference group is unrepresentative, users may evaluate themselves against an artificially elevated standard. Peers and near-peers may be especially influential because their similarity makes the comparison seem fair and the target’s advantage personally diagnostic. The relevant issue is thus not exposure alone, but locating oneself below a target who appears both superior and socially relevant. In the appearance domain, comparison with retouched targets makes a cultural ideal personally diagnostic by highlighting the discrepancy between one’s own appearance and the idealized target. Repetition can further stabilize this discrepancy as a habitual self-view, so that users begin to anticipate being judged against the same ideal by others. This provides a specific explanation for how social media exposure may be associated with appearance anxiety through upward comparison (11, 14, 15). Recent meta-analytic evidence likewise indicates that experimentally induced upward comparison has negative effects on body image and self-evaluation and that online social comparison is moderately associated with body-image concerns; recent appearance-anxiety research also reports a positive association between upward social comparison and appearance anxiety (8, 9, 17).

### 4.3 The mediating effect of envy

The findings further supported H3, showing a significant indirect association between social media use and appearance anxiety through envy. This interpretation is consistent with contemporary work distinguishing upward-motivating, relatively benign forms of envy from hostility-oriented malicious envy (25) and suggests that the observed indirect association requires an affective, rather than solely comparative, explanation. Cognitive appraisal theory suggests that another person’s advantage becomes emotionally consequential when it is relevant to personally important appearance goals (20). Beyond this theoretical account, an affective-motivational interpretation is also relevant because the five items primarily describe striving to attain another person’s advantage; the construct therefore more closely reflects achievement-oriented, relatively benign envy than hostility. When the valued ideal appears attainable, such striving may support constructive self-improvement; when the ideal is digitally enhanced or outside personal control, the same striving may coexist with tension and persistent appearance monitoring (39). For appearance anxiety, the relevant process is sustained self-evaluative attention. Envy can keep the comparison target and the perceived personal shortfall mentally active, thereby increasing monitoring of appearance, worry about inadequacies, and sensitivity to others’ judgments. The motivational direction of envy may also depend on perceived attainability and control. If the target appears realistically attainable, discomfort may be channeled into specific improvement goals; if the target is digitally enhanced or tied to attributes that are difficult to change, the same striving may become repetitive monitoring without resolution. This distinction helps explain why an achievement-oriented form of envy can coexist with anxiety: motivation to improve does not eliminate painful awareness of relative disadvantage. The observed association may therefore reflect persistent affective arousal and evaluative vigilance rather than hostility alone (26, 27). Accordingly, the result should not be interpreted as showing that all forms of envy are uniformly harmful. Moreover, the cross-sectional data do not show that social media use elicited envy; pre-existing appearance anxiety or other unmeasured characteristics may contribute to both experiences.

### 4.4 The chain mediating effects of upward social comparison and envy

The findings further supported H4, showing a significant chain mediation pattern involving upward social comparison and envy. This layered interpretation is also consistent with recent calls to integrate social-media affordances, sociocultural appearance pressures, comparison processes, and individual responses within contemporary body-image frameworks (12). Idealized social media content supplies a sociocultural standard; upward comparison converts that standard into a perceived personal discrepancy; envy gives the discrepancy emotional salience; and persistent emotional salience may heighten vigilance toward appearance-related evaluation. Each component therefore performs a different explanatory function. Exposure establishes what appears valued, comparison establishes where the self appears to stand, envy signals that the disadvantage matters, and appearance anxiety reflects concern about how the socially visible self will be judged. This sequence also explains why comparison and envy should not be collapsed into a single mediator: one is principally an evaluative cognition, whereas the other is an affective response that can prolong and intensify attention to the discrepancy. Rather than treating this sequence as a single-theory explanation, the pattern links different levels of analysis: social media use indexes an environment, upward comparison a relative self-evaluation, envy an affective response, and appearance anxiety a domain-specific social-evaluative concern. Compared with prior work on broader psychological outcomes, the present pattern identifies appearance anxiety as a specifically social-evaluative correlate. It suggests that the psychological relevance of the comparison–envy pattern depends not only on dissatisfaction with one’s appearance but also on anticipating that other people will use similar standards when evaluating the self. At the same time, the statistical sequence should be interpreted as one plausible organization of the associations rather than proof of a temporal process. The data do not show that these processes occurred in the proposed temporal order. Thus, the result describes an appearance-anxiety-specific chain pattern of statistical associations rather than a demonstrated causal process.

### 4.5 Implications

#### 4.5.1 Theoretical contributions

The theoretical contributions are most clearly understood in relation to three limitations in previous research: insufficient differentiation between the sociocultural source of appearance pressure and the psychological responses to it, frequent reliance on a single mediator, and greater attention to body dissatisfaction or general distress than to appearance-specific social-evaluative anxiety. This integration yields three specific contributions. First, by treating social media as a hybrid media-peer source of appearance ideals, the study distinguishes the sociocultural origin of appearance pressure from the psychological correlates of responding to that pressure. This is more precise than using social comparison as an omnibus explanation for the main association. Second, the tripartite influence model is applied as the overarching account of the main association, whereas social comparison theory and cognitive appraisal theory are restricted to the comparative and affective processes. This specifies a distinct explanatory role for each framework rather than treating them as interchangeable. It also adapts the tripartite model to a digital setting in which media and peer influences are no longer neatly separated: commercial images, influencers, peer presentations, and quantified feedback operate within the same platform environment. The contribution is therefore not a claim to test the full tripartite model, but a more precise conceptualization of social media as a hybrid source of sociocultural appearance pressure. The simultaneous observation of comparison-only, envy-only, and chain indirect-effect patterns further indicates that upward comparison and envy are related but not interchangeable correlates. Compared with single-mediator accounts, the joint model distinguishes the unique statistical roles of comparison and envy and shows that the two variables can be examined as both parallel and ordered correlates. This matters theoretically because exposure, comparative self-evaluation, and emotional response represent different levels of explanation. Treating them separately makes it possible to ask whether appearance anxiety is associated with cognitive discrepancy, affective salience, or their combined pattern rather than assuming one undifferentiated mechanism. Third, the domain-specific contribution is the explicit treatment of appearance anxiety as a social-evaluative outcome. Body dissatisfaction primarily concerns negative evaluation of one’s own body, whereas appearance anxiety additionally concerns how the self is expected to be evaluated by other people. Examining this outcome therefore connects sociocultural appearance standards not only to private dissatisfaction but also to anticipated scrutiny, rejection, and interpersonal uneasiness, extending prior research focused largely on body dissatisfaction, depression, or general well-being (7, 21, 22). Because appearance anxiety centers on anticipated negative evaluation by others, it represents a more focused social-evaluative outcome than general body dissatisfaction or psychological distress. The residual direct association also indicates that comparison and envy do not exhaust the relationship and points to additional processes, such as appearance-ideal internalization and appearance-focused use, for future study.

#### 4.5.2 Practical implications

The findings further clarify the pattern of associations linking social media use to appearance anxiety among university students. The chain mediation findings suggest that interventions in higher education should address not only the amount of time spent on social media but also appearance-focused content, automatic comparison habits, and envy following comparison. The findings have the following implications for aesthetic education, mental health education, and digital media governance.

First, digital media aesthetic literacy should be systematically incorporated into aesthetic education curricula. Aesthetic education should extend beyond traditional art appreciation to address university students’ mediated aesthetic experiences. Media literacy interventions can help alleviate body image concerns (40), and programs for adolescents have shown potential to reduce shape- and appearance-related concerns (41). Universities can design instruction around how images are produced, filtered, and recommended, helping students recognize how filters, beauty enhancement, retouching, posed photography, and commercial marketing shape appearance standards and how algorithmic recommendations can lead to repeated exposure and aesthetic narrowing.

Second, diverse aesthetics and body appreciation should be used to counter narrow appearance standards. A major social foundation of appearance anxiety is the continual reinforcement of “template-based beauty.” Aesthetic education can expose students to diverse representations of gender, body type, age, skin tone, physical condition, and cultural tradition and encourage them to understand beauty in terms of bodily function, personal qualities, life experience, and cultural meaning, thereby reducing the tendency to treat appearance as the sole measure of self-worth. Experimental research has shown that exposure to body-positive content can improve positive affect, body satisfaction, and body appreciation among young women, although it may also increase self-objectification. Related content should therefore avoid turning “diverse bodies” into mere objects of visual consumption (42). When promoting diverse aesthetics, universities should simultaneously emphasize agency, functionality, and personal value.

Third, tiered psychological interventions should target the “comparison–envy” chain. Mental health education can help students identify triggers of automatic upward comparison, such as repeatedly viewing appearance-focused influencers, checking likes and comments, or evaluating themselves after editing selfies. For students with strong comparison tendencies or marked appearance concerns, cognitive reappraisal, attentional shifting, rumination reduction, and self-acceptance training may help weaken the absolutist belief that “if others are more attractive, I am not good enough.” Envy does not inevitably lead to negative outcomes and can, under appropriate conditions, be transformed into motivation for self-improvement (39). The key is to help students distinguish attainable growth goals from uncontrollable appearance standards and translate post-comparison emotions into concrete and healthy action without self-devaluation.

Fourth, a collaborative aesthetic support environment involving universities, families, platforms, and society should be developed. Universities can reduce implicit appearance-based judgment in counseling and student activities and improve referral and support pathways for appearance anxiety. Families should place greater emphasis on abilities, effort, and personal character. Media platforms should increase the visibility of authentic, diverse, and minimally edited images and optimize content prompts, youth modes, and recommendation mechanisms to reduce continual reinforcement of idealized appearance content. Collaborative governance should focus not simply on restricting social media use but on improving the content environment and evaluative climate encountered by university students so that they can maintain a critical distance from aesthetic information while using these platforms.

Fifth, tiered identification and continuous evaluation mechanisms should be established. Indicators such as appearance comparison, sensitivity to appearance evaluation, and appearance-focused social media activity may be appropriately included in orientation programs, mental health screening, and routine counseling, while avoiding the pathologization of normal appearance concerns. Universal media literacy education may be sufficient for most students; group counseling and individual consultation can be provided for those with strong comparison tendencies or high appearance anxiety; and students with marked social avoidance, disordered eating, or emotional distress should be referred promptly to professional services. The effectiveness of courses and interventions should also be evaluated through pretests and posttests, follow-up surveys, and process feedback so that practical recommendations become part of a sustainable and verifiable support system rather than one-off campaigns.

### 4.6 Limitations and future directions

This study has several limitations.

First, it used a cross-sectional design, and all variables were measured at a single time point using self-report questionnaires. The temporal order of the variables therefore cannot be established, and reverse effects of appearance anxiety on social media use and comparison behavior cannot be ruled out. Although experimental and longitudinal evidence suggests that exposure to appearance ideals may harm body image, the overall longitudinal association is relatively small (43). Future studies could use multiwave longitudinal designs, cross-lagged models, or randomized experiments to examine more rigorously the causal direction and dynamic development of the “social media use–upward comparison–envy–appearance anxiety” pathway. Ordered indirect effects can also be inflated in cross-sectional models, and unmeasured common causes may account for part of the observed pattern. Accordingly, the terms ‘mediation’ and ‘chain mediating effect’ refer here to statistical indirect effects rather than a demonstrated causal chain (33).

Second, all core variables were based on self-reports from the same participants and may therefore have been affected by social desirability, recall bias, and common method variance. Poor fit of the one-factor model indicates only that a single common factor cannot account for all covariance and does not fully rule out common method bias. Future research could combine smartphone screen-use records, platform behavior logs, experience sampling, and interview data and improve the robustness of the evidence through temporal separation or multimethod measurement.

Third, social media use was treated as a global construct. The study did not distinguish active posting from passive browsing, appearance-focused from non-appearance-focused use, or short-video platforms, image-based communities, and acquaintance-based social networking platforms. Because appearance-focused comparison may be more closely related to body image disturbance than general social media use (44), future research should differentiate platforms, content, and use patterns and examine specific behaviors such as algorithmic recommendations, filter use, and selfie editing. Moreover, the adapted measure originated from a Facebook-intensity scale. Platform affordances such as algorithmic feeds, short-video immersion, filters, and interaction norms may differ in the Chinese social media ecosystem, so platform-specific validity remains to be established.

Fourth, the measures require further contextual refinement. The upward social comparison measure combined items from different questionnaires, while the envy measure did not fully distinguish dispositional from situational envy or benign from malicious envy. Future research could develop or validate more context-specific measures for appearance-related social media experiences and test measurement invariance across gender, educational level, and cultural groups. Although all HTMT ratios were below 0.85, future research should continue to examine measurement invariance and platform-specific validity.

Fifth, convenience sampling was used. Participant age was not collected for analysis, so the age range and mean could not be reported and age could not be included as a covariate. Although participants were recruited from multiple universities, representativeness remained limited by the distribution of institutions, regional composition, and self-selection. The model also did not test the boundary conditions associated with gender, body appreciation, self-esteem, media literacy, or self-compassion. Future research could use stratified random sampling and multigroup analysis to identify the individuals and contexts most susceptible to upward comparison and envy.

## 5 Conclusion

This study examined the relationship between social media use and appearance anxiety among university students in China and the roles of upward social comparison and envy in this relationship. The findings reveal a coherent associational pattern in which greater social media use is associated with higher levels of appearance anxiety, while upward social comparison and envy contribute both independently and jointly through a chain mediation pattern. Taken together, these results suggest that appearance-related concerns in digital environments may arise from the interplay of multiple processes: a sociocultural context that heightens the salience of appearance standards, comparative self-evaluation against superior targets, and affective responses to perceived disadvantage. By distinguishing the broader social-media context from the cognitive process of upward comparison and the affective role of envy, this study provides a more differentiated framework for understanding appearance anxiety. These findings may also offer useful implications for aesthetic education, media literacy, and mental-health support in higher education.

## Data Availability

The original contributions presented in the study are included in the article/supplementary material. Further inquiries can be directed to the corresponding authors.

## Appendix A

### Scale items

The items below are English translations of the items administered in this study. All items used the 5-point Likert response format.

#### Social media use scale (6 items)

Using social media apps is part of my everyday activities.

I feel somewhat proud when I tell other people which social media app I use most often.

Logging in to the social media app I use most often has become a daily habit.

I feel lonely when I have not logged in to the social media app I use most often for a while.

I feel that I am part of the community on the social media app I use most often.

I would be sorry if the social media app I use most often were shut down.

#### Upward social comparison scale (6 items)

When evaluating my current life or appearance, I often compare myself with people on social media who are better off than I am.

When things do not go well for me, I often think about people on social media who are doing better than I am.

When evaluating my current life and studies, I often compare myself with people on social media who are doing better than I am.

When evaluating my interpersonal skills, I prefer to compare myself with people whose interpersonal skills are stronger than mine.

Whenever I consider whether I am capable of doing something, I often compare myself with people who are more capable than I am.

On the social media platform I use most often, I frequently compare myself with people who are more successful than I am.

#### Envy scale (5 items)

When I envy someone, I think about how I can achieve the same level of success in the future.

If I find that someone else is better than I am, I try to improve myself.

Envying others motivates me to achieve my goals.

I strive to achieve the same level of excellence as others.

When I see another person’s superior qualities, achievements, or possessions, I strive to attain similar qualities, achievements, or possessions myself.

#### Social appearance anxiety scale (10 items)

I worry that people think I am unattractive.

I worry that others will evaluate my appearance negatively.

I feel very uncomfortable when others comment on my appearance.

I worry that I have missed many opportunities because of my appearance.

I worry that people talk behind my back about flaws in my appearance.

I feel nervous when having my photograph taken with others.

I worry about my ability to attract members of the opposite sex.

I feel uneasy when people are clearly looking at me.

Because I worry about my appearance, I feel tense when talking with others.

I want to change my appearance.
